# Supplementary material for: Understanding FBA Solutions under Multiple Nutrient Limitations
Source: Metabolites. 2021 Apr 21;11(5):257. doi: 10.3390/metabo11050257 (PMC8143296; doi:10.3390/metabo11050257)
Supplement: Supplementary file 1 [file metabolites-11-00257-s001.zip › Results/iJR904_2/EFM_yield_analysis/oxygen-5/cost_plotoriginal_with_hidden_metabolites_M_glc__D_e_M_o2_e.pdf]

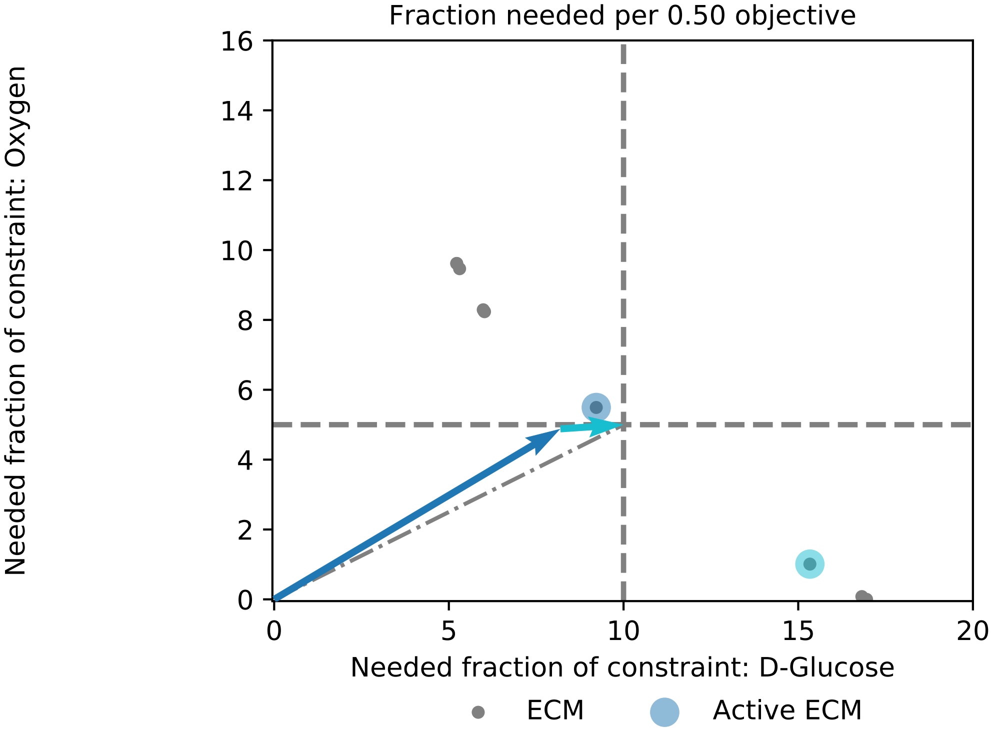
Fraction needed per 0.50 objective
Needed fraction of constraint: Oxygen
Needed fraction of constraint: D-Glucose
0
2
4
6
8
10
12
14
16
0
5
10
15
20
ECM
Active ECM
