# Supplementary figures and images for: Understanding FBA Solutions under Multiple Nutrient Limitations
Source: Metabolites. 2021 Apr 21;11(5):257. doi: 10.3390/metabo11050257 (PMC8143296; doi:10.3390/metabo11050257)

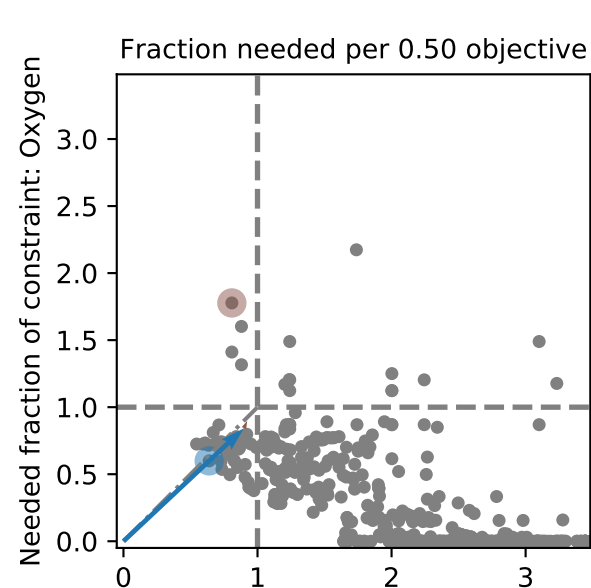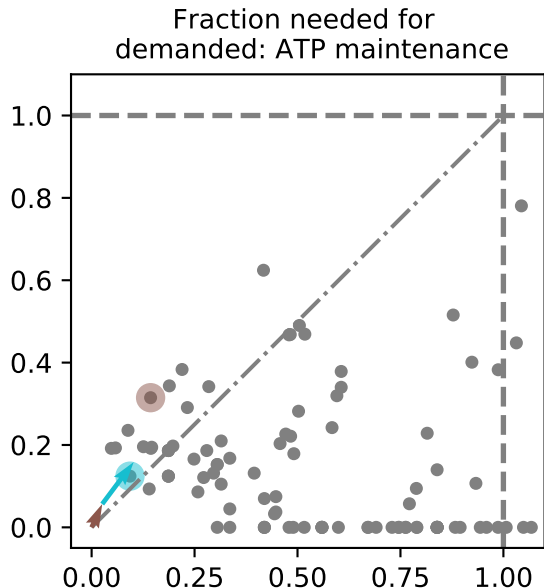

Needed fraction of constraint: D-Glucose

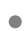

ECM

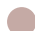

Active ECM

Supplement: Supplementary file 1 [file metabolites-11-00257-s001.zip › Results/e_coli_core_2/EFM_yield_analysis/With_ATPm_two_constr/cost_plotoriginal_network_M_glc__D_e_M_o2_e.pdf]

● Relative constraint usage in ECM    — Used fraction of constraints

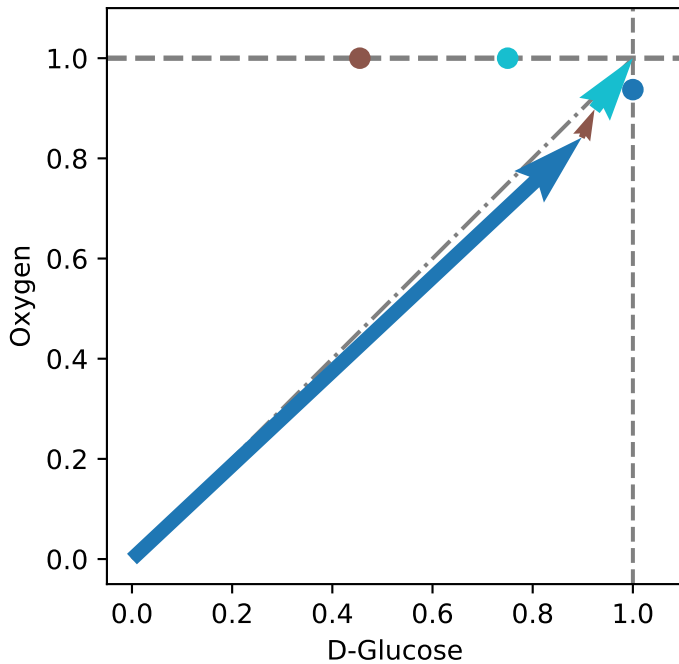

ECM ID

● 62    ● 108    ● 227

Supplement: Supplementary file 1 [file metabolites-11-00257-s001.zip › Results/e_coli_core_2/EFM_yield_analysis/With_ATPm_two_constr/cost_plot_ECMs_one_figureoriginal_network.pdf]

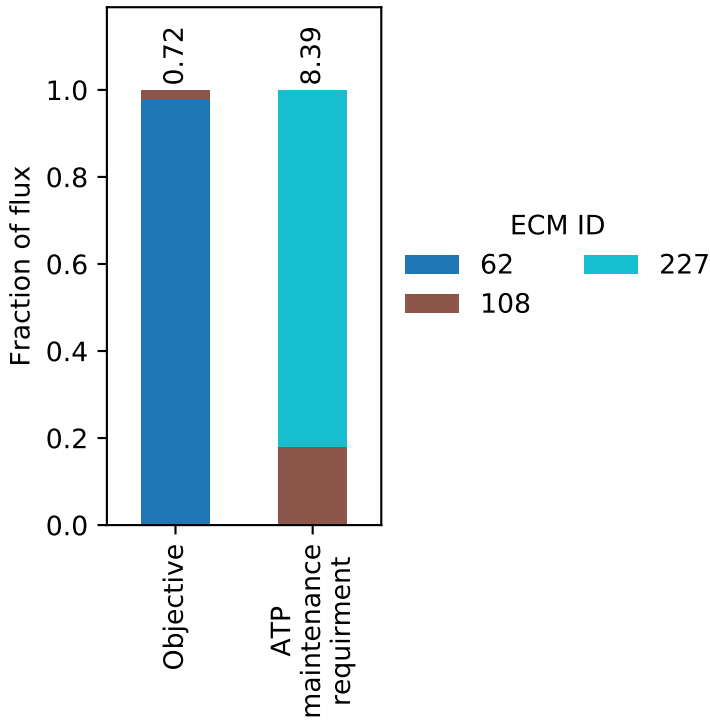

Supplement: Supplementary file 1 [file metabolites-11-00257-s001.zip › Results/e_coli_core_2/EFM_yield_analysis/With_ATPm_two_constr/plot_ECM_fractionsoriginal_network.pdf]

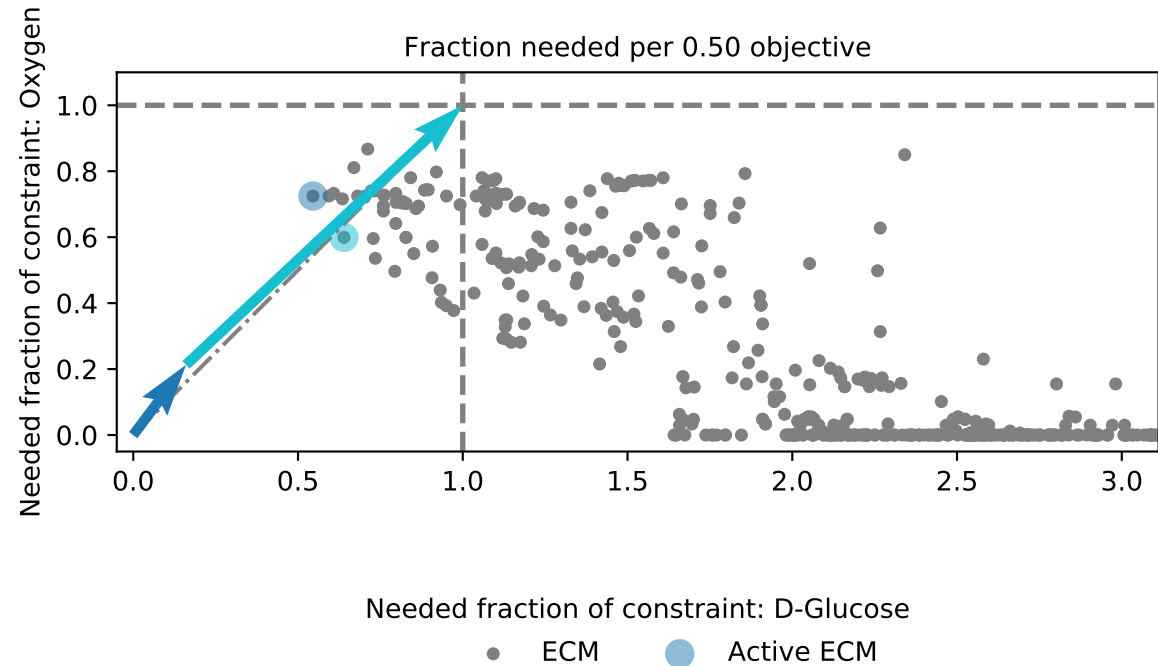

Supplement: Supplementary file 1 [file metabolites-11-00257-s001.zip › Results/e_coli_core_2/EFM_yield_analysis/wo_ATPm/cost_plotoriginal_network_M_glc__D_e_M_o2_e.pdf]

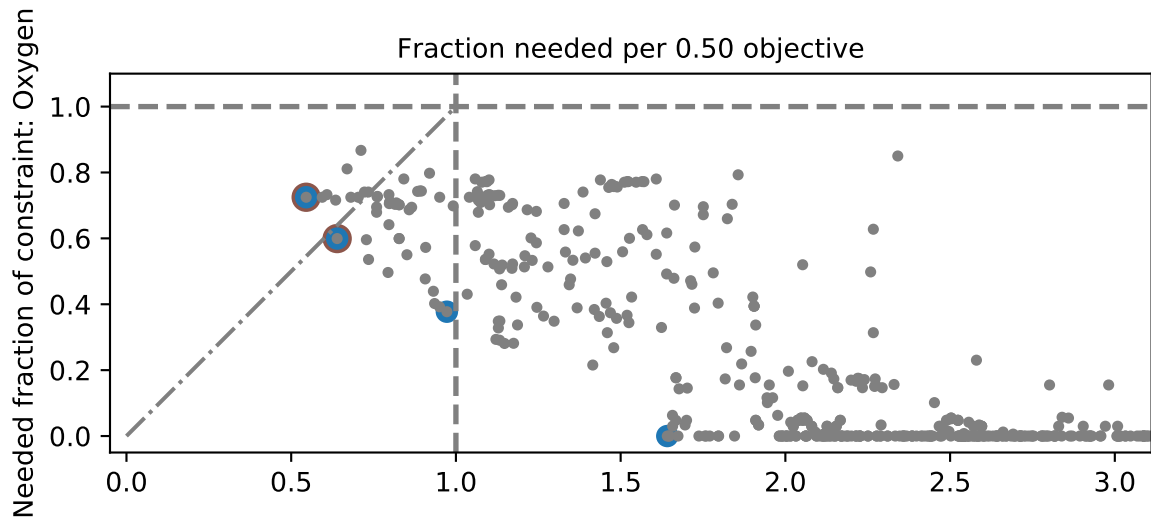

Supplement: Supplementary file 1 [file metabolites-11-00257-s001.zip › Results/e_coli_core_2/EFM_yield_analysis/wo_ATPm/various_approaches_constraints_M_glc__D_e_M_o2_e.pdf]

Needed fraction of constraint: Oxygen

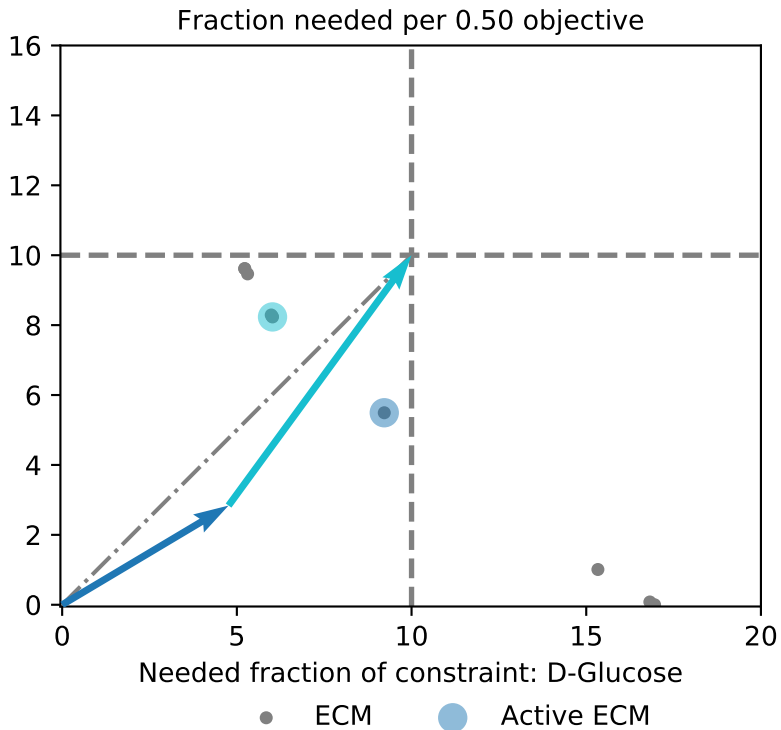

Needed fraction of constraint: D-Glucose

● ECM ● Active ECM

Supplement: Supplementary file 1 [file metabolites-11-00257-s001.zip › Results/iJR904_2/EFM_yield_analysis/oxygen-10/cost_plotoriginal_with_hidden_metabolites_M_glc__D_e_M_o2_e.pdf]

Needed fraction of constraint: Oxygen

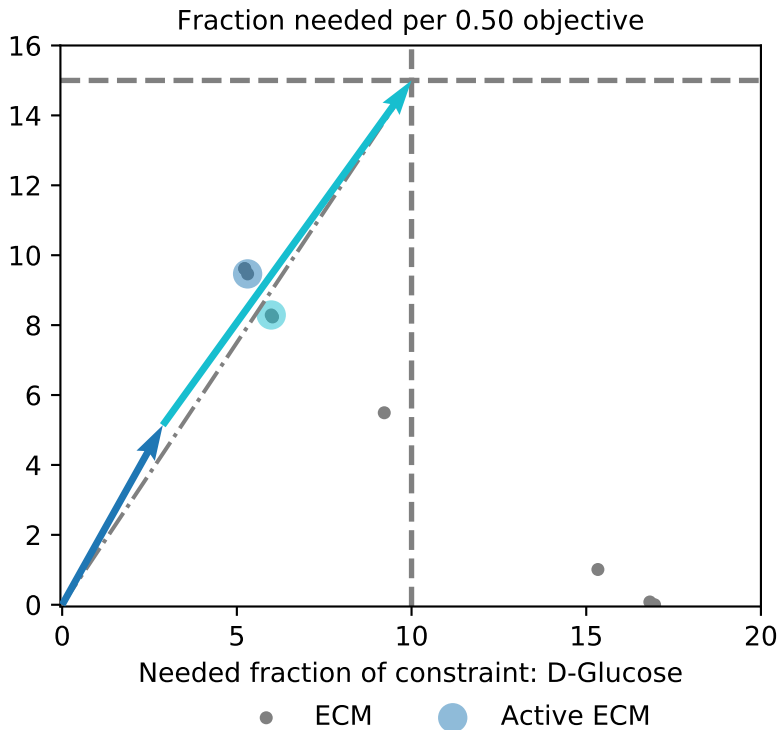

Supplement: Supplementary file 1 [file metabolites-11-00257-s001.zip › Results/iJR904_2/EFM_yield_analysis/oxygen-15/cost_plotoriginal_with_hidden_metabolites_M_glc__D_e_M_o2_e.pdf]

Product formation per 0.5 unit biomass

Oxygen per glucose

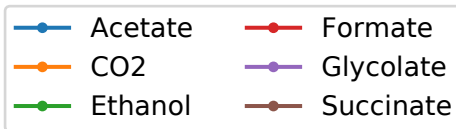

Supplement: Supplementary file 1 [file metabolites-11-00257-s001.zip › Results/iJR904_2/EFM_yield_analysis/product_formation_per_ox_per_glc.pdf]

● Relative constraint usage in ECM — Used fraction of constraints

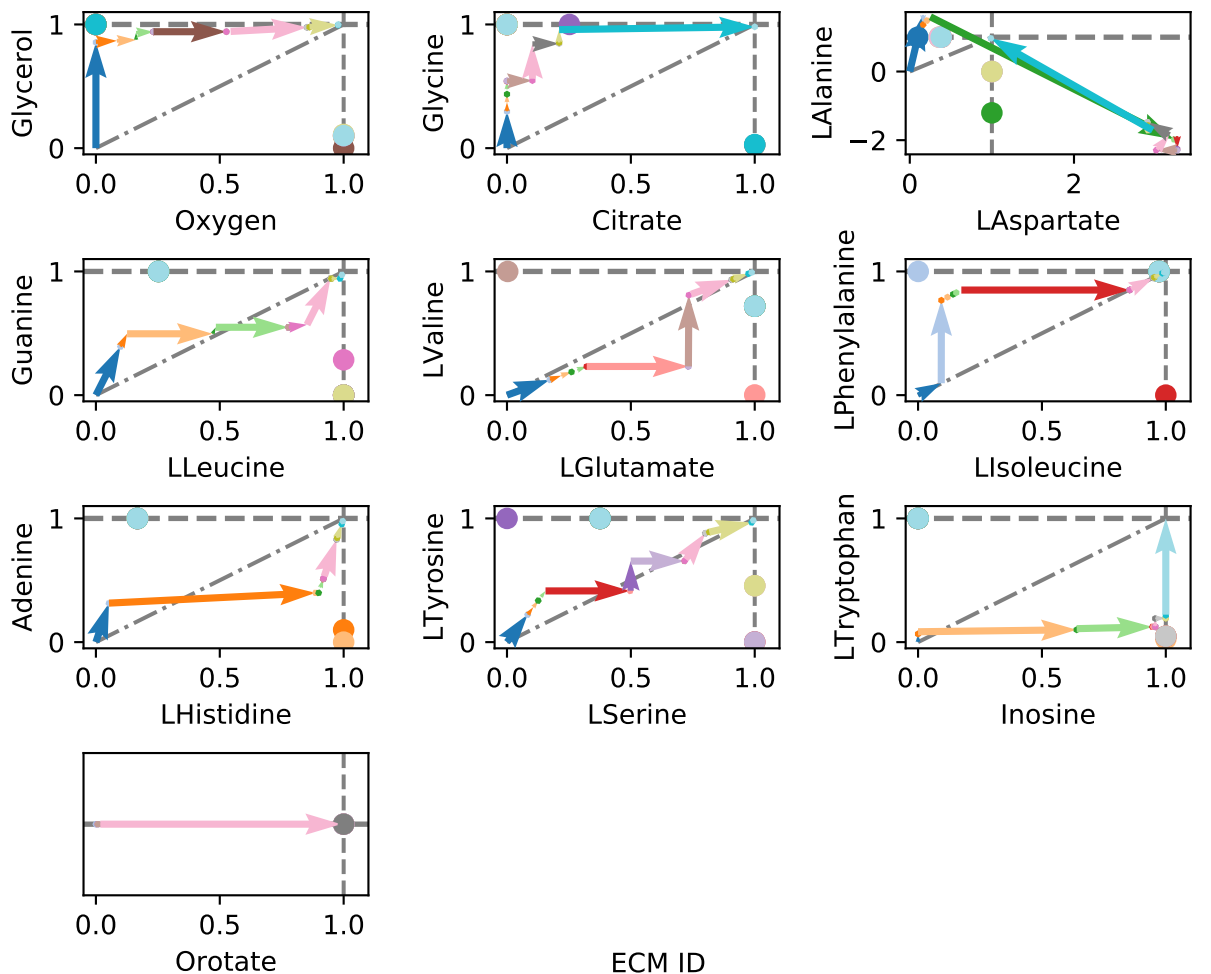

|     |      |      |      |      |      |
|-----|------|------|------|------|------|
| 98  | 1044 | 2660 | 3476 | 4260 | 5241 |
| 689 | 1222 | 2800 | 3836 | 4354 | 5497 |
| 972 | 1473 | 3127 | 4204 | 4659 | 5559 |
| 996 | 1656 |      |      |      |      |

Supplement: Supplementary file 1 [file metabolites-11-00257-s001.zip › Results/Lactobacillus_plantarum_WCFS1_Official_23_May_2019_18_45_01_2/EFM_yield_analysis/all/cost_plot_ECMs_one_figureactive_network_with_hidden_metabolites.pdf]

● Relative constraint usage in ECM      — Used fraction of constraints

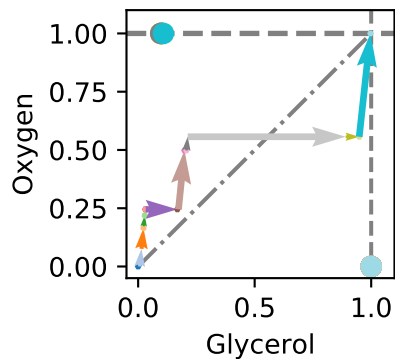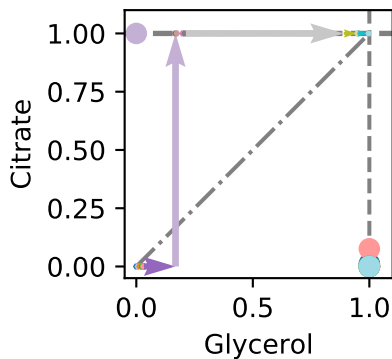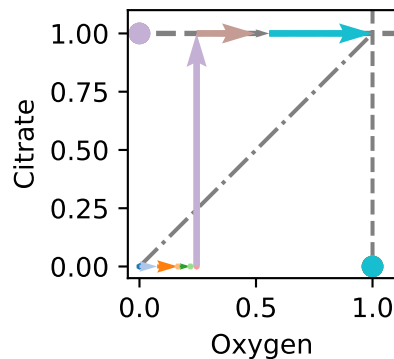

ECM ID

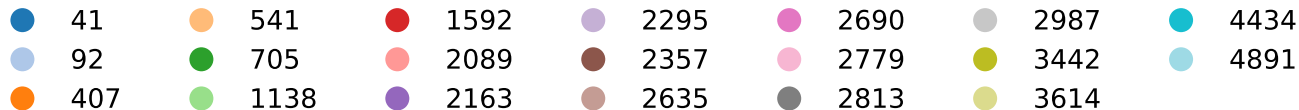

Supplement: Supplementary file 1 [file metabolites-11-00257-s001.zip › Results/Lactobacillus_plantarum_WCFS1_Official_23_May_2019_18_45_01_2/EFM_yield_analysis/glyc_cit_o2/cost_plot_ECMs_one_figureactive_network_with_hidden_metabolites.pdf]

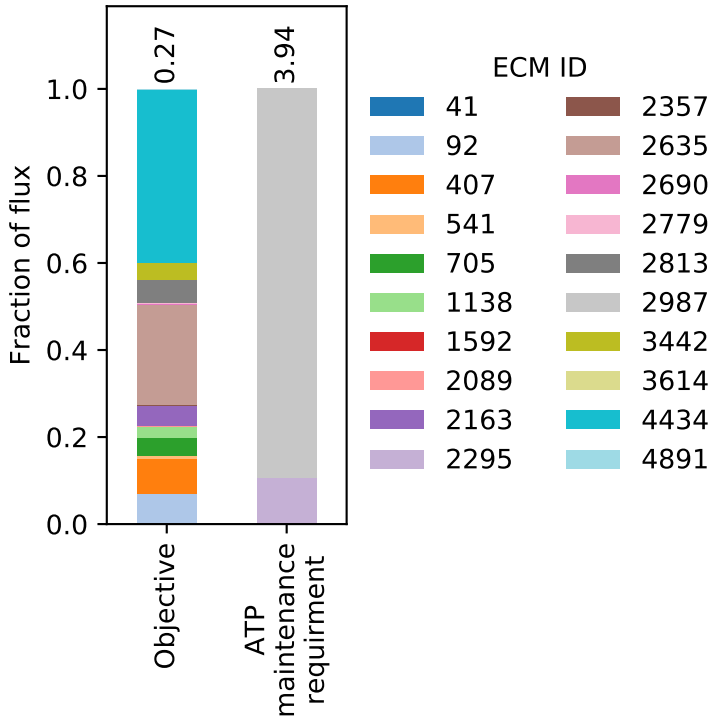

Supplement: Supplementary file 1 [file metabolites-11-00257-s001.zip › Results/Lactobacillus_plantarum_WCFS1_Official_23_May_2019_18_45_01_2/EFM_yield_analysis/glyc_cit_o2/plot_ECM_fractionsactive_network_with_hidden_metabolites.pdf]

● Relative constraint usage in ECM

— Used fraction of constraints

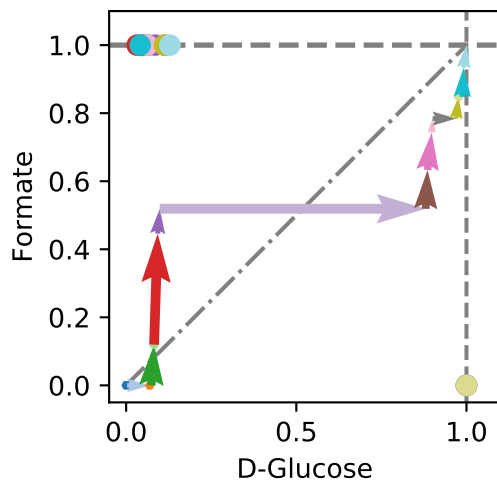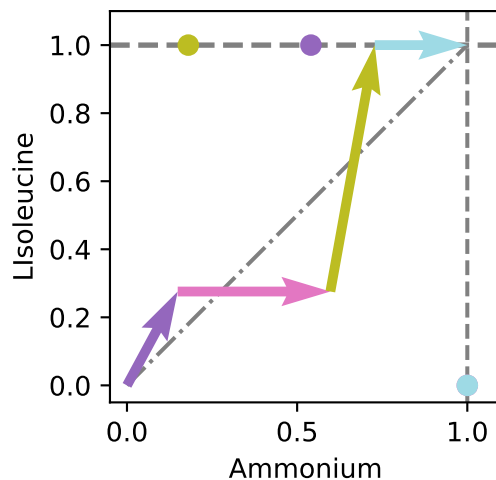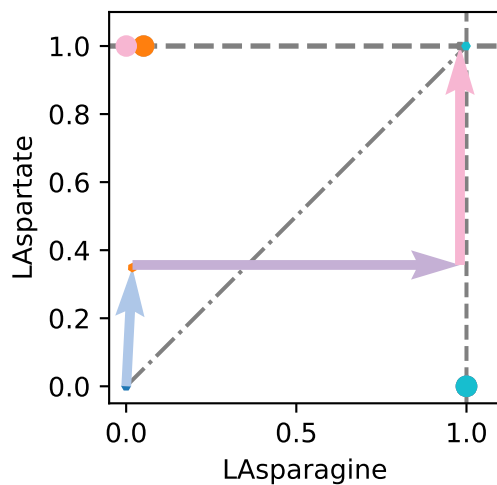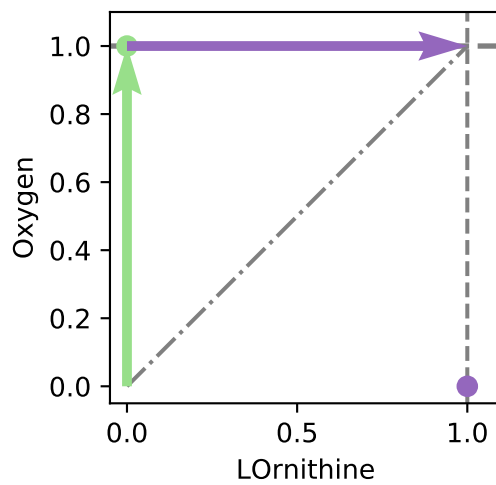

ECM ID

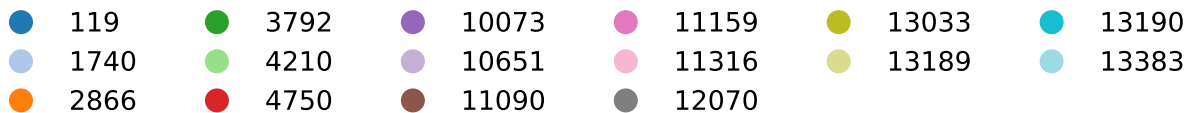

Supplement: Supplementary file 1 [file metabolites-11-00257-s001.zip › Results/MG1363_20190628_2/EFM_yield_analysis/FBA_adjusted/cost_plot_ECMs_one_figureactive_network_with_hidden_metabolites.pdf]

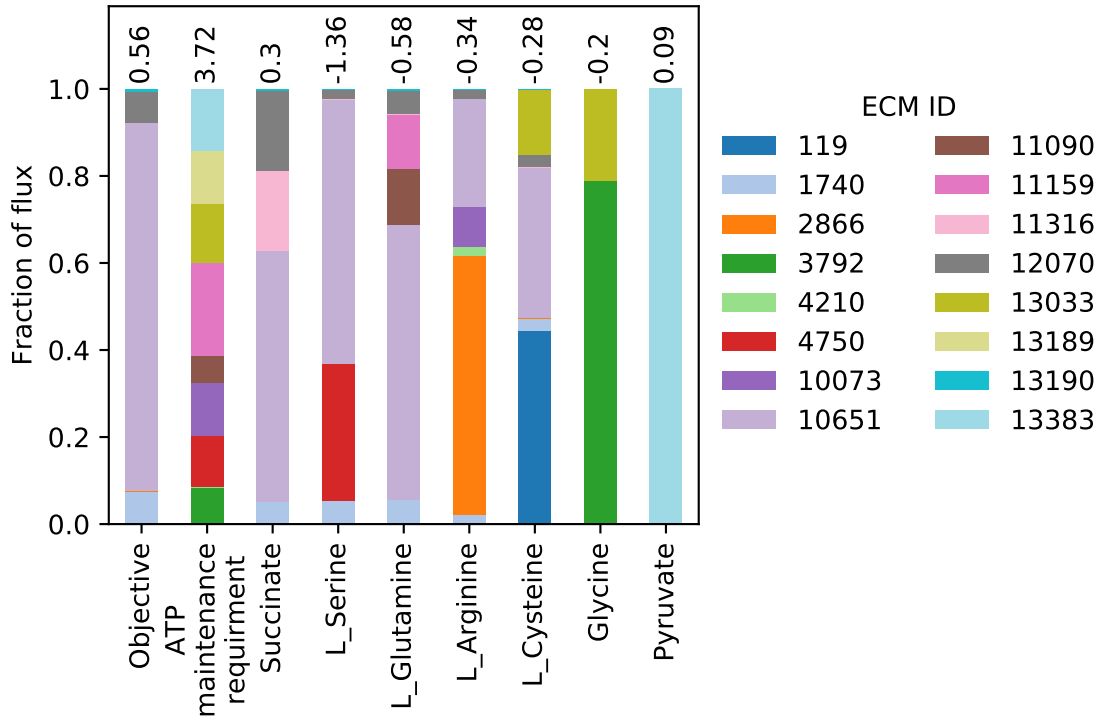

Supplement: Supplementary file 1 [file metabolites-11-00257-s001.zip › Results/MG1363_20190628_2/EFM_yield_analysis/FBA_adjusted/plot_ECM_fractionsactive_network_with_hidden_metabolites.pdf]
